# Contribution of 2 D strain in the detection of subtle myocardial involvement in group A and B patients with chronic obstructive pulmonary disease

**DOI:** 10.3389/fmed.2025.1471588

**Published:** 2025-06-04

**Authors:** Rania Kaddoussi, Ikram Chamtouri, Wafa Dhouib, Imen Touil, Saoussen Ben Abdallah, Monia Daami, Fatma Ezzahra Elassoufi, Walid Jomaa, Wissal Rouetbi, Ahmed Turki, Khaldoun Ben Hamda

**Affiliations:** ^1^Department of Pneumology, Hospital Fatouma Bourguiba, Monastir, Tunisia; ^2^Department of Cardiology B, Hospital Fatouma Bourguiba, Monastir, Tunisia; ^3^Department of Preventive Medicine, Hospital Fatouma Bourguiba, Monastir, Tunisia; ^4^Department of Medical, Moknine Hospital, Monastir, Tunisia

**Keywords:** chronic obstructive, heart failure, spirometry, walk test, strain

## Abstract

**Background:**

Myocardial involvement mediated by chronic obstructive pulmonary disease (COPD) is a common cause of morbidity and mortality. Conventional transthoracic echocardiography (TTE) parameters are poor in the detection of subclinical myocardial dysfunction.

**Aim:**

To investigate the contribution of strain in the early detection of cardiac damage in clinically stable COPD patients.

**Methods:**

This was a comparative study between COPD patients (classified A or B) with normal and reduced right ventricle (RV) strain. The COPD assessment test (e.g., CAT score), spirometry [e.g., forced expiratory volume in 1 s (FEV_1_, L)], 6 min walk test [e.g., 6 min walk distance (6MWD, m)], and both conventional TTE [i.e., left ventricular ejection fraction (LVEF), right atrium (RA), RV, left ventricle global longitudinal strain (LV GLS)], and strain (e.g., impaired RV strain is > −19), were performed.

**Results:**

Eighty COPD patients [mean ± standard deviation (SD): age = 66 ± 9 years, LVEF = 60.1 ± 5%, RA = 25 ± 7%, RV = −19.9 ± 3.7%, LV GLS v −21.1 ± 2, and 48% had impaired RV strain] were included. Compared to COPD patient with normal RV strain, those with reduced RV strain had (i) Lower 6MWD (310 ± 113 vs 470 ± 104 m; *p* = 0.001), (ii) Lower FEV_1_ (1.63 ± 0.73 vs 2.18 ± 0.41 L; *p* = 0.012), and (iii) Higher CAT score (21 ± 10 vs 13 ± 6; *p* = 0.012). An impaired RV strain was associated with a higher risk of hospitalizations for acute exacerbation in the post inclusion year, (respectively for 55% and 25%; *p* = 0.024). No death was recorded during the study period.

**Conclusion:**

Group A and B COPD patients having normal conventional TTE parameters, speckle tracking is a key parameter in the detection of subclinical myocardial dysfunction.

## Introduction

Chronic obstructive pulmonary disease (COPD) is a public health problem worldwide (1). It is one of the main causes of high morbidity and mortality (2). The development of heart failure during COPD is a major predictive factor of exacerbation, hospital readmission, and mortality (3). Both right- and left-sided heart failure could frequently be noted in COPD patients (4). Several studies have revealed that this comorbidity is related, on the one hand, to the structural and physiological changes in the pulmonary vascularization predisposing to the right heart failure even before pulmonary hypertension (PH) (5) and, on the other hand, to the common cardiovascular risk factors, such as smoking, chronic systemic inflammation, and endothelial dysfunction that are leading factors of heart failure (6). Subtle heart failure has no symptomatic repercussion on COPD with overlapping symptoms, such as dyspnea, leading to an under diagnosis of incipient myocardial damage (7). Right (RV) and left (LV) ventricles subclinical dysfunctions remain challenging (8). The conventional echocardiographic measurements are poor in detecting subtle myocardial injury (4). Speckle tracking study, a technique of myocardial deformation measurement, provides promising results in the early detection of LV and RV dysfunction (9, 10).

Thus, the main objective of the current study was to identify subtle RV and LV dysfunction using two-dimensional strain in clinically stable COPD patients. The secondary aim was to evaluate the effect of altered RV free wall strain on the risk of hospital readmission.

## Materials and methods

### Study design

This was an observational cross-sectional study conducted over a period of 17 months (from January 2023 to May 2024) in the Cardiology B department at Fattouma Bourguiba Hospital, Monastir, Tunisia, in patients diagnosed with group A or B COPD.

### Study population

Patients’ recruitment was carried out by two Tunisian teams from the departments of pulmonology (Moknine hospital, Monastir, Tunisia) and cardiology B department (Fattouma Bourguiba hospital, Monastir, Tunisia). Patients aged more than 18 years and with confirmed diagnosis of group A or B COPD (2, 10). The following non-inclusion criteria were applied: evidence of LV ejection fraction (LVEF) < 50% on echocardiography, severe valvular heart disease (11), pulmonary embolism, PH, coronary artery disease, conduction abnormalities, atrial fibrillation on electrocardiogram, contraindications for the 6 min walk test (6MWT) (12), and COPD exacerbations 3 months before enrollment (13). Files of patients with abnormalities noted while performing echocardiography were excluded from final analysis.

### Sample size calculation

The sample size was determined based on the comparison of two independent groups: patients with normal versus reduced right ventricular (RV) strain. Using data from Botelho et al. (14), the mean global right ventricular longitudinal strain (RVGLS) was −21.2% ± 4.4% in the control group and −17.2% ± 4.4% in COPD patients with RV dysfunction, yielding an absolute effect size (Δ) of 4%. The sample size calculation was performed using the two-sample *t*-test formula (15):


$$n=2{\left(Z\alpha /2+Z\beta \right)}^{2}\sigma^{2}/{\text{Δ}}^{2}$$

where α = 0.05 (Z = 1.96), power = 80% (Z = 0.84), standard deviation (σ) = 4.4%, and Δ = 4%. Based on this, a minimum of 38 patients (19 per group) was required. To account for an estimated 20% dropout rate, the final recommended sample size was increased to 46 patients (23 per group).

### Variables and data collection

Chronic obstructive pulmonary disease diagnosis was confirmed by a spirometry using the Global Initiative for Chronic Obstructive Lung Disease (GOLD) criteria, indicating a non-reversible ventilatory deficit [i.e., post-bronchodilator ratio between forced expiratory volume in 1 s and forced vital capacity (FEV_1_/FVC) < 0.70] (16). COPD group A patients are defined as having ≤ 1 mMRC (modified Medical Research Council) dyspnea scale or CAT score (the COPD assessment test) < 10 with a history of ≤ 1 exacerbation per year and not leading to hospital admission (16). And COPD group B patients are defined as having ≥ 2 mMRC dyspnea scale or CAT score ≥ 10 with a history of ≤ 1 exacerbation per year and not leading to hospital admission (16).

During the study period, patients filled out a questionnaire written in the local Arabic dialect. The questionnaire had three components and its estimated duration is 20 min. The first part involved the patients’ social and demographic characteristics (e.g., age, sex, medical history). The second part included COPD data (e.g., treatment, number of hospitalizations, exacerbation). The third part involved the (CAT) test (15), including questions about eight areas, to assign an overall score ranging from 0 to 40. Higher scores indicates that COPD has a greater impact on the patient’s health and wellbeing (17).

After filling out the questionnaire and on the same day, spirometry and 6 min walk test (6MWT) were done. Spirometry was performed by an experienced technician using a portable spirometer (SpirobankG MIR, del Maggiolino 12500155 Roma, Italy) according to the international recommendations (18). Spirometry data [e.g., FVC (L), FEV_1_ (L), FEV_1_/FVC ratio (absolute value)], were reported as absolute values and as percentage of predicted values (19). The 6MWT was conducted on a flat, straight corridor and the patients were required to walk as far as possible for 6 min to calculate the 6 min walk distance (6MWD, m) (20). The directions given to the patients throughout the test were in compliance with international guidelines (12). The predicted 6MWD was calculated according to local norms (20)

A conventional trans-thoracic echocardiography (TTE) was performed on a second day, using a Vivid E9 echocardiography system (General Electric Medical System). The same operator performed all TTEs to limit inter-operator variations (21). All conventional TTE parameters were performed, including LV diameters, wall thickness and volumes (22). Left ventricle ejection fraction (LVEF) was estimated using the Simpson method (22). Peak mitral E and A waves in pulse Doppler, e’ wave in tissue Doppler imaging, E/e’ ratio as well as the left atrial area (LAA) and volume were measured (22). Right ventricle (RV) function was evaluated using peak of RV systolic myocardial velocity (S wave) and tricuspid annular plane systolic excursion (TAPSE) (22). The right atrial area and LAA and volume were measured. Systolic pulmonary artery pressure (sPAP) was calculated on the peak of tricuspid regurgitation (22). Speckle tracking analyses using Echopac software version 112 and automated functional imaging (AFI) was used to evaluate both LV and RV strain, and RA reservoir function. A RA strain < 25% is considered impaired (23). An altered RV free wall strain is > −19 (24) and an impaired LV global longitudinal strain (LV GLS) is > −20 (25).

A total of 1 year after TTE performing, all patients were contacted by telephone to check the number of hospitalizations during this year and deaths.

### Ethical consideration

The study was approved by the medical and research ethics committee at Fattouma Bourguiba University Hospital (Approval number IORG 0009738 N 160 OMB 0990-0279). All patients signed an informed consent to participate in this study.

### Statistical analysis

Quantitative data were expressed as mean ± standard deviation (SD), and categorical data as percentages. The normality of distribution was verified using the Kolmogorov-Smirnov test. The Chi-square test and Fisher’s exact test were appropriately used for categorical variables and percentage comparisons. The Student’s *t*-test was utilized to compare the means of quantitative variables. Values were considered significant when p was ≤ 0.05. The partial eta-squared effect size was calculated, and Hedge’s values were used for effect size measurement (26). An effect size of ≤ 0.2 was described as a small effect, around 0.5 as a medium effect, around 0.8 as a large effect, and more than 1.30 as very large effect (26).

A univariate analysis was initially conducted. The association between dependent (i.e., RV Strain) and each independent variable (i.e., age, sex, living habits, smoking, FEV_1_, 6MWD, CAT score and echocardiographic parameters) was analyzed to include variables that could be highly predictive *a priori* in a multivariate analysis model.

During multivariate analysis, variables with *p* < 0.25 were included in the multivariate model and they were analyzed using backward stepwise logistic regression. The latter included all selected variables and progressively removed those that did not contribute sufficient information to the model at each step. Thus, only the independent variables remained in the final step. Variables with a significant Odds Ratio (OR, *p* ≤ 0.05) still present in the final step were considered significant independent variables in the observed multivariate model. To determine the association between quantitative variables, correlation was used to determine the correlation coefficient (r) and regression analysis was used to study the regression equation: Y = a + b X, in which Y is dependent, X is independent, b is slope, and a is intercept.

All statistical analyses were performed using SPSS (Statistical Package for the Social Sciences) version 21.0 IBM.

## Results

**FIGURE 1:**
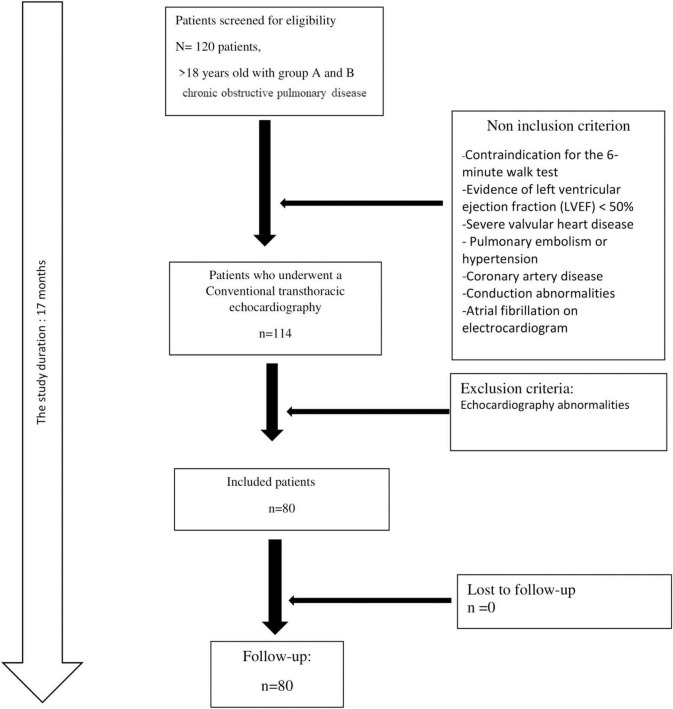
The flow chart of the study population.

A total of 80 patients with a mean ± SD age of 66 ± 9 years were included (Figure 1). Male predominance was noted (83.3%). The number of current smokers was 29 (36.2%). The mean ± SD FEV_1_ value was 1.95 ± 0.64 L (62 ± 20%). Using the CAT score, nine patients were scored > 30 (Table 1).

**TABLE 1:** Associated factors with a reduced right ventricle (RV) strain in groups A and B patients with COPD.

Characteristics	Total population (*N* = 80)	Normal RV strain (*N* = 32)	Impaired RV strain (*N* = 48)	*P*-value	Effect size
Age (years)	66 ± 9	65 ± 8	66 ± 9	0.87	0.4
Sex (male)	67 (83.8)	24 (24/32)	43 (43/48)	0.083	–
Living habits	–				
Current smoker	29 (36.2)	17 (17/32)	12 (12/48)	0.005	–
FEV_1_ (L)	1.95 ± 0.64	2.18 ± 0.41	1.63 ± 0.73	0.012	0.7
FEV_1_ (%)	62 ± 20	69 ± 13	51 ± 23	0.012	0.7
6MWD (m)	396 ± 121	470 ± 104	310 ± 113	0.001	0.4
6MWD (%)	56 ± 18	66 ± 15	43 ± 16	0.001	0.4
CAT	16.9 ± 9	13 ± 6	21 ± 10	0.012	0.3
< 10	24 (30.0)	16 (16/32)	8 (8/48)	–	–
10–20	30 (37.5)	16 (16/32)	14 (14/48)		–
21–30	17 (21.3)	0 (0.0)	17 (17/48)		–
> 30	9 (11.3)	0 (0.0)	9 (9/48)		–

Data were mean ± standard deviation or number (%). CAT, COPD assessment test; COPD, chronic obstructive pulmonary disease; FEV_1_, forced expiratory volume in 1 s; 6MWD, 6 min walk distance.

**FIGURE 2:**
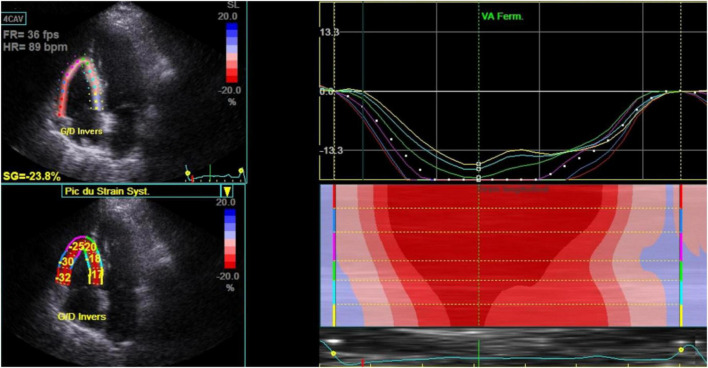
Right ventricle strain in stable chronic obstructive pulmonary disease patients (*n* = 80). 4 CAV, 4 cavities; Bpm, beats per minute; Ferm VA, fermeture valve aortique; Fps, frames per second; FR, frame rate; HR, heart rate; SG, global strain; SL, lateral strain.

**FIGURE 3:**
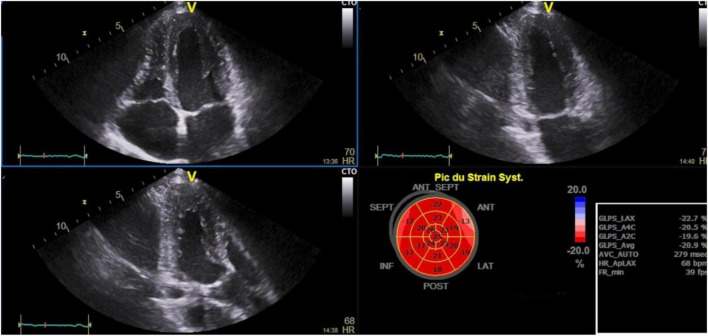
Left ventricle global longitudinal strain and bull’s eye in stable chronic obstructive pulmonary disease patients (*n* = 80). ANT, anterior; ANT-SEPT, antero-septal; GLPS-A4C, global longitudinal peak strain 4 cavities; GLPS_LAX, global longitudinal peak strain long axis; GLPS-A2C, global longitudinal peak strain 2 cavities; GLPS-AVG, global longitudinal peak strain average; HR, heart rate; INF, inferior; LAT, lateral; POST, posterior; SEPT, septal.

Mean ± SD of LVEF, RA reservoir, RV strain (Figure 2), LV GLS (Figure 3) were 60.7 ± 5.1%, 24.5 ± 6.6%, −19.9 ± 3.7%, and −21.1 ± 2.4.

Among the 80 patients, 48 had impaired RV strain, with lower 6MWD (*p* = 0.001) and FEV_1_ (*p* = 0.012), and a higher CAT score (*p* = 0.012) compared to those with normal RV strain with a medium size effect (Table 1).

Univariate analysis revealed a significant association between damaged RV strain and FAC, S wave RV, TAPSE, and sPAPS (Table 2).

**TABLE 2:** Univariate analysis: Association between conventional echocardiographic parameters and reduced right ventricle (RV) strain in COPD patients.

Characteristics	Total population (*N* = 80)	Normal RV strain (*N* = 32)	Reduced RV strain (*N* = 48)	*P*-value	Effect size
LV ejection fraction (%)	60.7 ± 5.1	60.75 ± 4.819	60.77 ± 9.599	0.986	0.16
FAC (%)	45.4 ± 7.7	50.343 ± 5.839	42.166 ± 7.143	0.000	0.51
TAD (mm)	27.7 ± 2.7	27.593 ± 2.949	27.854 ± 2.576	0.677	0.16
RVBD (mm)	32.3 ± 3.6	32.593 ± 3.653	32.208 ± 3.649	0.645	0.14
RVMD (mm)	24.4 ± 2.5	23.875 ± 2.485	24.750 ± 2.621	0.140	0.13
RVLD (mm)	62.5 ± 5.7	60.875 ± 6.282	63.583 ± 5.089	0.037	0.20
RAA (cm^2^)	12.6 ± 2.1	12.531 ± 2.361	12.693 ± 2.041	0.744	0.15
S wave RV (cm/s)	12.8 ± 2.1	13.906 ± 1.956	12.062 ± 1.803	0.000	0.40
TAPSE (mm)	20.8 ± 3.28	22.937 ± 3.426	19.500 ± 2.352	0.000	0.40
sPAP (mmHg)	32.8 ± 6.4	29.000 ± 5.364	35.375 ± 5.796	0.000	0.51
RV wall thickness (mm)	6.6 ± 0.8	6.343 ± 1.035	6.708 ± 0.682	0.061	0.18
IVC (mm)	14.9 ± 1.3	14.66 ± 1.537	15.17 ± 1.226	0.104	0.08
E/A	0.98 ± 0.29	1.060 ± 0.290	0.938 ± 0.282	0.065	0.17
LAA (cm^2^)	14.5 ± 2.4	14.931 ± 2.597	14.333 ± 2.364	0.290	0.15
IVS thickness (mm)	9.0 ± 0.8	8.906 ± 0.928	9.208 ± 0.742	0.111	0.05
LV EDD (mm)	45.8 ± 3.4	45.312 ± 3.335	46.229 ± 3.520	0.248	0.15
LV PW (mm)	8.7 ± 0.9	8.625 ± 0.975	8.791 ± 0988	0.460	0.01

Data were mean ± standard deviation. COPD, chronic obstructive pulmonary disease; SD, standard deviation; FAC, fractional area change, IVC, inferior vena cave; IVS, inter ventricular septum; LAA, left atrial area; LV, left ventricle; LVEDD, left ventricle end diastolic diameter; LVEF, left ventricle ejection fraction; PW: posterior wall; RA, right atrium; RAA, RA area; RV MCD, RV mid cavity diameter; RVBD, RV basal diameter; RVLD, RV longitudinal diameter; sPAP, systolic pulmonary artery pressure; TAD, tricuspid annulus diameter; TAPSE, tricuspid annular plane systolic excursion.

The multivariate analysis identified three factors associated with the reduction of RV strain. The sPAPS was found to be a significant factor for damaged RV strain, with an adjusted OR of 1.2 (*p* = 0.001) (Table 3).

**TABLE 3:** Multivariate analysis: factors associated with the changes in right ventricle (RV) strain [*n* = 80 chronic obstructive pulmonary disease (COPD)].

Characteristics	Adjusted odds ratio	95% confidence interval		*P*-value
6 min walk distance (m)	0.985	0.978	0.993	0.001
systolic pulmonary artery pressure (mmHg)	1.214	1.079	1.366	0.000
S wave RV (cm/s)	0.526	0.338	0.818	0.004

**FIGURE 4:**
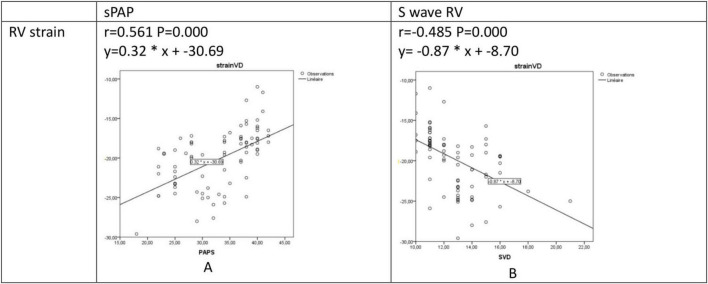
Regression and correlation analysis between RV strain and sPAPS **(A)** and S wave RV **(B)**. RV, right ventricular: S wave, right ventricular systolic myocardial velocity; sPAP, systolic pulmonary artery pressure.

Alteration in RV strain was (i) Positively correlated with sPAPS (r = 0.561, *P* = 0.000, Figure 4A), and (ii) Negatively correlated with S wave RV (r = −0.485, *P* = 0.000, Figure 4B).

Compared to the group with normal RV strain, the group with altered RV strain was associated with a higher risk of hospitalizations for acute exacerbation in the post inclusion year (%) (25 vs. 55, respectively; *p* = 0.024) with a small size effect.

During the study period, no death was recorded.

## Discussion

Right heart failure is a major cause of mortality and morbidity in patients with COPD. The prognosis of these patients can be affected by prompt diagnosis, effective therapy, and careful observation (4).

This study highlighted that stable COPD without PH could cause subtle LV and RV dysfunction before developing symptoms of heart failure. Progressive cardiovascular impairment related to COPD increases the mortality and morbidity rates (27). According to previous studies, the possible mechanisms leading to this damage are chronic multisystemic inflammation, high oxidative stress, high levels of inflammatory markers such as TNF-α, interleukins, and C-reactive protein, endothelial alteration, and the interaction between the heart and the lungs (4, 5, 20). Cardiovascular damages include pulmonary hypertension, and left and right heart failure (28). Heart failure is a major cause of mortality in patients with COPD (1). Initially, ventricular dysfunction is asymptomatic or oligosymptomatic, especially for the right ventricle in the early stage of COPD without pulmonary hypertension (29). Cardiac involvement in patients with recurrent exacerbations in group E has already been documented (6, 30, 31). According to Freixa et al., nearly one in every eight COPD patients requiring hospitalization develop severe RV dysfunction 3 months following the initial exacerbation (32). However, cardiac involvement is underdiagnosed in group A and B patients with COPD (33). Chronic hypoxemia and low blood oxygen levels can engender pulmonary hypertension or elevated blood pressure in the pulmonary arteries through several mechanisms (6, 7). Chronic hypoxemia can be undiagnosed for a long time in COPD patients since it is initially limited to intensive exertion and can be hidden by adaptive processes, especially in group A patients with low dyspnea manifestations. Classifying the cause of respiratory problems among patients with both conditions can be challenging. Systematic screening for cardiac involvement is therefore beneficial. Indeed, early diagnosis of subtle left and right dysfunction could change the therapeutic strategy and the prognosis of COPD patients (14).

As it is available and non-invasive, echocardiography is currently used to diagnose the effects of COPD on the heart. However, the parameters of standard echocardiography are not effective in screening subtle cardiac damage. Several studies have revealed the geometry of the LV, the function changes encompassing systolic and diastolic dysfunction, the hypertrophy of LV, and the reduced volumes (34). However, systolic and diastolic LV function could be totally normal in standard echocardiography parameters as shown in the present study. Speckle tracking is a promising method for the early detection of LV dysfunction and evidence gathered over the last decade has shown GLS to be more sensitive to left ventricular dysfunction than LVEF and to provide additional prognostic information (35). Pizarro et al. showed a significant damage in LV GLS in COPD patients with a reduced regional strain in the apical and septal walls that is correlated with COPD severity (8). In this study, despite the normal parameters of LV standard echocardiography, subclinical LV alteration was revealed by GLS. RV dysfunction in COPD patients is associated with worse outcomes and an increased mortality (3). Previous studies have shown that RV hypertrophy, dilatation, and systolic dysfunction are common in COPD patients regardless of pulmonary hypertension and the increased RV afterload (36, 37). These findings may be due to an elevated pulmonary vascular resistance and a reduced pulmonary artery/arterial compliance (38). RV remodeling develops early during COPD, leading to subclinical RV dysfunction (38). RV GLS is a powerful parameter in RV in subclinical dysfunction secondary to chronic respiratory diseases, such as COPD and fibrotic interstitial lung diseases (39). Speckle tracking echocardiography allows the quantification of RV dysfunction and the screening of discrete and localized contractile loss (39). The current study demonstrated that despite normal RV systolic parameters (S wave RV, TAPSE), RV free wall strain was damaged. Right atrium exercise intolerance may also be altered by heart dysfunction (6). Therefore, in the case of reduced 6MWD, a subclinical cardiac involvement should be screened, especially in non-exacerbated COPD. A high CAT score was also noted in patients with altered RV Strain (*p* = 0.012). Since many factors contribute to the impact of the quality of life in individuals with COPD, the significance of cardiac involvement is often neglected, particularly in groups A and B (12). This emphasizes how crucial it is to provide COPD patients with global management plans that take into account both cardiac and pulmonary issues even in group A and B patients with COPD. The quality of life of such patients can be enhanced by integrating care approaches that take into account the complex character of these illnesses. Besides, cardiovascular disorders are correlated with a higher risk of hospitalizations as shown in this meta-analysis where right heart failure was a potential risk factor for the 30 days readmission of COPD patients (40). COPD patients with heart failure have a far higher risk of being hospitalized, which exacerbates their already complicated medical needs. Early detection of heart failure plays a crucial role in reducing the hospitalization rates and improving patient outcomes. Therefore, an early identification of heart failure enables prompt intervention, which can slow the evolution of the disease and improve symptoms.

### Study strengths and limitations

The study has two notable strengths. The study was conducted in an outpatient unit in a low-income nation, especially Tunisia. Second, the sample size was computed, which improved the study’s statistical robustness One of the limitations of the present study is the absence of magnetic resonance imaging as a gold standard for the diagnosis of RV and LV subtle dysfunction as well as the short-term follow-up and the lack of data. Moreover, LV and RV strain could be affected by other factors beyond COPD. Indeed, despite the high sensitivity of speckle tracking in the early detection of cardiac involvement, its low specificity, its reliance on operator experience, on good image quality, and on frame rate (41) as long as the intervendor differences in the accuracy of detecting regional functional abnormalities (42) and its dependence on loading conditions and extrinsic mechanical factors, particularly anterior chest wall deformity and/or pectus excavatum (43) remain a major limitation.

## Conclusion

Cardiac damage is a common complication in COPD patients. It could worsen the prognosis and increase mortality. Due to the overlapping symptoms of cardiac failure and COPD, myocardial damage is often overlooked. Based on this study, LV and RV strain could detect silent myocardial involvement at early stages. Close follow-up using speckle tracking allows the detection of subtle cardiac damage and the indication of the appropriate strategy to prevent advanced myocardial dysfunction.

## Data Availability

The original contributions presented in this study are included in this article/supplementary material, further inquiries can be directed to the corresponding author.
